# Effects of snow properties on humans breathing into an artificial air pocket – an experimental field study

**DOI:** 10.1038/s41598-017-17960-4

**Published:** 2017-12-15

**Authors:** Giacomo Strapazzon, Peter Paal, Jürg Schweizer, Markus Falk, Benjamin Reuter, Kai Schenk, Hannes Gatterer, Katharina Grasegger, Tomas Dal Cappello, Sandro Malacrida, Lukas Riess, Hermann Brugger

**Affiliations:** 1 Institute of Mountain Emergency Medicine, Eurac Research, Bolzano, Italy; 2 Department of Anaesthesiology and Intensive Care Medicine, Hospitallers Brothers Hospital, Teaching Hospital of the Paracelsus Medical University Salzburg, Salzburg, Austria; 3 Department of Perioperative Medicine, Barts Heart Centre, William Harvey Research Institute, Barts & The London School of Medicine&Dentistry, Queen Mary University of London, London, UK; 4 WSL Institute for Snow and Avalanche Research SLF, Davos, Switzerland; 5 Department of Sport Science, Medical Section, University of Innsbruck, Innsbruck, Austria; 6 Department of Anaesthesiology and Intensive Care Medicine, Medical University Innsbruck, Innsbruck, Austria

## Abstract

Breathing under snow, e.g. while buried by a snow avalanche, is possible in the presence of an air pocket, but limited in time as hypoxia and hypercapnia rapidly develop. Snow properties influence levels of hypoxia and hypercapnia, but their effects on ventilation and oxygenation in humans are not fully elucidated yet. We report that in healthy subjects breathing into snow with an artificial air pocket, snow density had a direct influence on ventilation, oxygenation and exhaled CO_2_. We found that a rapid decline in O_2_ and increase in CO_2_ were mainly associated with higher snow densities and led to premature interruption due to critical hypoxia (SpO_2_ ≤ 75%). However, subjects in the low snow density group demonstrated a higher frequency of test interruptions than expected, due to clinical symptoms related to a rapid CO_2_ accumulation in the air pocket. Snow properties determine the oxygen support by diffusion from the surrounding snow and the clearance of CO_2_ by diffusion and absorption. Thus, snow properties are co-responsible for survival during avalanche burial.

## Introduction

Asphyxia, i.e. hypoxia and hypercapnia, is the primary cause of death from snow avalanche^1^. Approximately 70% of completely buried avalanche victims (i.e. head and chest below the snow) die of asphyxia within 35 minutes^2,3^. A retrospective study in Canada has suggested that asphyxia occurs earlier in avalanche burials in a maritime climate, where snow density is higher, compared to those in a continental climate^4^. However, several studies have shown that breathing under a snow layer is possible in the presence of a patent airway with or without an air pocket (i.e. any space in front of mouth and nose)^5–9^. Previously, an experimental field study demonstrated that the volume of the air pocket closely determines the speed of oxygen desaturation; specifically, a 1 L air pocket compared to 2 L led to an ealier onset of hypoxaemia^8^. However, a recent study showed that breathing into snow with a patent airway but no air pocket in front of the upper airway was possible for a short time with an exceedingly high ventilatory effort^6^. In addition, completely buried subjects could maintain adequate oxygenation and ventilation, breathing into an artificial air pocket device. This device separates carbon dioxide (CO_2_)–rich exhaled air from oxygen (O_2_)–rich inhaled air, thus avoiding excessive increases of inspiratory CO_2_ with progressive life-threatening hypoxia and hypercapnia^9^. Apart from the size of the potential air pocket, previous hypotheses regarding survival during avalanche burial have considered that the development of hypoxeamia in avalanche victims could also depend upon specific snow properties^8,9^.

Snow is a highly porous media containing high amounts of interstitial air (e.g. 67% air with a snow density of 300 kg/m^3^, a snow density that it is commonly found in deposits of dry snow avalanches)^10,11^. Previous studies suggested that snow density seems to affect ventilation and survival chances of avalanche victims^4,8,9^. Thus, the aim of this experimental field study was to elucidate the effects of snow properties on the development of critical levels of hypoxia and hypercapnia in subjects breathing into an artificial air pocket; specifically focusing on the effect of differing snow densities and other snow properties on ventilation and oxygenation in humans. We hypothesised that the speed of the onset of critical physiological levels of hypoxia and hypercapnia would be dependent upon differences in specific snow properties.

## Results

### Subjects

Fourteen males were enrolled in the study and all subjects were included in the final data analysis. Specifically, twelve study subjects underwent three test series whilst breathing into artificial air pockets of fixed volume (4 L), in different snow densities. The mean age of the study subjects was 33.8 ± 7.3 years, weight 78.2 ± 8.1 kg, height 179 ± 5.3 cm. At baseline testing, forced expiratory volume in 1 second (FEV_1_) and forced vital capacity (FVC) were 4.2 ± 0.5 L and 5.3 ± 0.4 L, respectively. Two additional subjects, matched by age (36 and 34 years), weight (68 and 78 kg), height (178 and 179 cm), FEV_1_ (4.1 and 4.8 L) and FVC (5.0 and 6.2 L), were recruited as controls and required to breath into an airtight 4 L control plastic bag (i.e. void of additional gas or gas exchange) in each one of the three test series.

### Test interruptions

**Figure 1:**
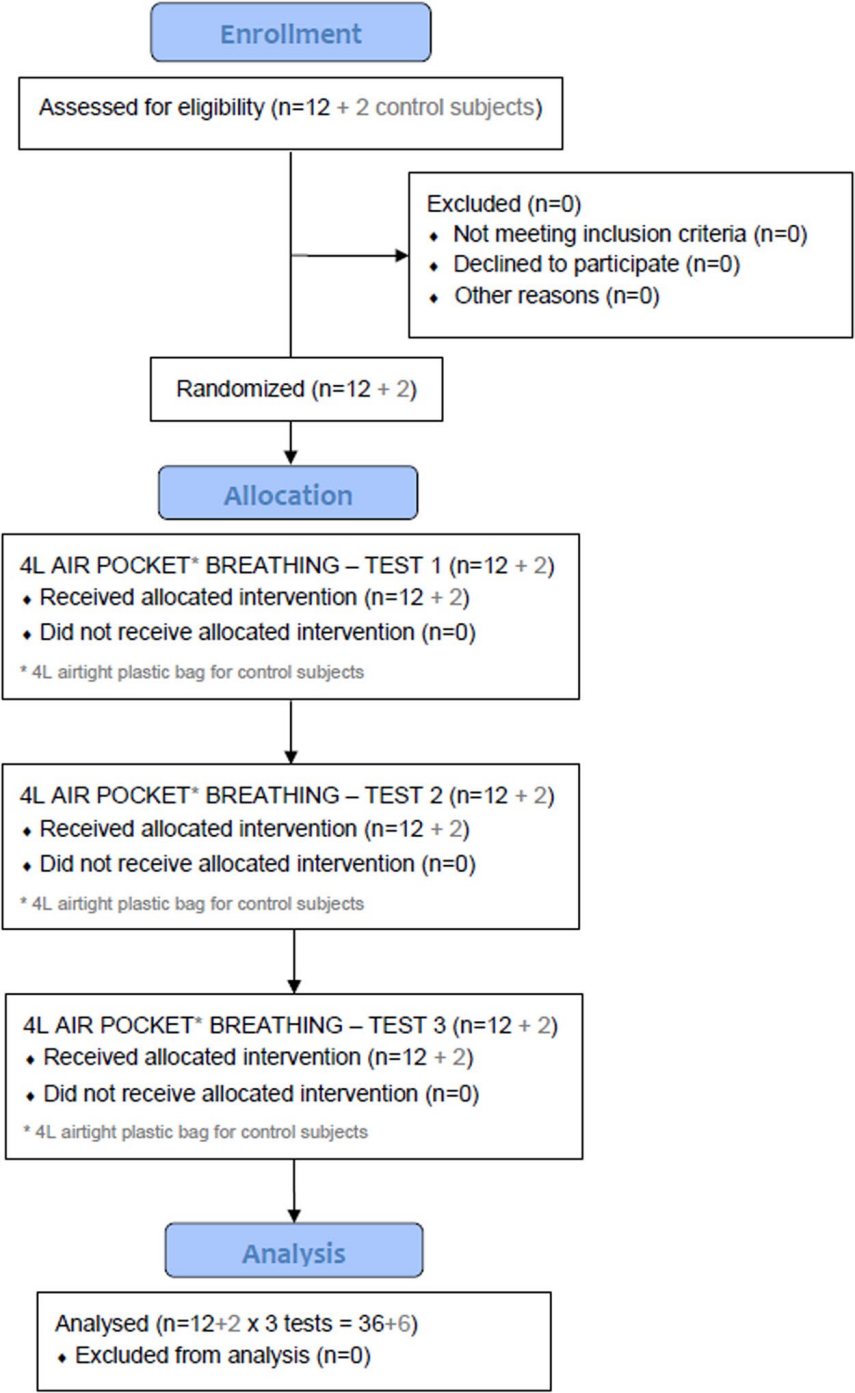
Flow diagram of the study with enrollment, allocation and analysis of the subjects (12 study subjects + 2 control subjects).

All subjects were requested to breathe up to 30 min; in total 36 study tests and 6 control tests were performed (Fig. 1). Specifically, within study tests 18 out of 36 tests lasted 30 min; 13 tests were terminated prematurely due to evident hypoxaemia (peripheral oxygen saturation SpO_2_ ≤ 75%), plus 5 tests were interrupted at the subject’s request due to clinical symptoms (dyspnea (n = 3), dizziness (n = 1), and dyspnea and headache (n = 1)). Interruption time and reason, SpO_2_, end-tidal carbon dioxide (etCO_2_), O_2_ and CO_2_ concentration in the air pocket (O_2_ pocket and CO_2_ pocket) for each subject in each test series are shown in Table 1. Within control tests, all 6 tests were terminated prematurely due to critical levels of hypoxaemia (SpO_2_ ≤ 75%).

**Table 1:** Interruption time, difference from baseline to minimum value (Δ min) of SpO_2_ and O_2_ pocket and difference from baseline to maximum value (Δ max) of etCO_2_ and CO_2_ pocket per subject and test.

Subject	Test series 1							Test series 2							Test series 3						
	Interruption time (min: s)			Δ min SpO_2_* (%)	Δ max etCO_2_* (mmHg)	Δ min O_2_ pocket (%)	Δ max CO_2_ pocket (%)	Interruption time (min: s)			Δ min SpO_2_* (%)	Δ max etCO_2_* (mmHg)	Δ min O_2_ pocket (%)	Δ max CO_2_ pocket (%)	Interruption time (min: s)			Δ min SpO_2_* (%)	Δ max etCO_2_* (mmHg)	Δ min O_2_ pocket (%)	Δ max CO_2_ pocket (%)
1	30	0		−4	na	−8.20	4.99	11	0	(a)	−22	14	−11.49	5.90	13	0	(a)	−25	21	−11.56	5.92
2	30	0		−6	na	−7.39	4.57	30	0		−4	7	−7.68	4.65	8	6	(b)	−12	35	−11.16	6.03
3	9	30	(a)	−25	13	−10.06	5.36	10	36	(a)	−28	14	−11.29	5.63	10	5	(a)	−24	16	−12.19	5.90
4	5	55	(a)	−21	5	−7.93	4.11	7	0	(a)	−25	7	−9.31	4.95	5	55	(a)	−24	16	−9.50	4.40
5	30	0		−15	18	−9.70	5.05	8	30	(a)	−27	18	−10.39	4.69	7	3	(a)	−25	14	−12.66	5.46
6	30	0		−2	12	−7.49	4.77	30	0		−3	12	−7.70	4.93	12	15	(b)	−22	22	na	na
7	30	0		−12	7	−10.26	5.48	23	11	(b)	−21	37	−11.39	6.14	8	20	(a)	−27	19	−10.09	5.76
8	30	0		−12	18	−9.99	5.79	30	0		−21	11	−11.38	6.12	11	18	(a)	−25	20	−12.33	5.75
9	30	0		−9	9	−8.60	4.56	30	0		−6	11	−7.61	4.81	30	0		−13	11	−11.09	5.00
10	30	0		−9	22	−9.30	4.93	24	20	(b)	−19	34	−10.99	6.08	11	12	(a)	−23	25	−11.76	5.74
11	30	0		−5	4	−5.69	3.85	17	11	(b)	−20	23	−7.99	4.87	30	0		−9	12	−9.51	5.32
12	30	0		−7	19	−8.06	4.22	30	0		−16	42	−10.59	5.82	30	0		−9	21	−10.09	5.52
Ctrl 1	3	30		−22	54	nn	nn	3	20		−27	21	nn	nn	3	48		−29	29	nn	nn
Ctrl 2	3	12		−24	15	nn	nn	3	8		−40	16	nn	nn	2	50		−24	44	nn	nn

(a) indicates interruption due to SpO_2_ ≤ 75% and (b) interruption requested. *For control subjects difference from baseline to stop value. Ctrl, control; etCO_2_, end tidal CO_2_; SpO_2_, peripheral oxygen saturation. na, value not available due to technical failure. nn, value not measured due to the experimental protocol.

### Physical snow properties and gas concentration in the air pocket

Snow density, permeability, coefficient of variation (COV) of penetration resistance, standard deviation (SD) of penetration resistance, and snow temperature per subject and test are shown in Table 2. Median snow density was 364 kg/m^3^ (range 155–481 kg/m^3^), and in accordance with the study protocol, varied between test days. Median SD of the penetration resistance was 1.6 N (range 0.33–11 N). Snow temperatures within the artificial avalanche deposit ranged from −5 to 0 °C before tests and were related to environmental conditions; snow temperature around the cavity increased by approximately 0.5 to 1 °C during each test.

**Table 2:** Snow density, permeability, coefficient of variation (COV) of penetration resistance, standard deviation (SD) of penetration resistance and snow temperature per subject and test.

Subject	Test series 1					Test series 2					Test series 3				
	Snow-density (kg/m³)	Permeability (10^−10^ m^2^)	COV penetration resistance (N)	SD penetration resistance (N)	Snow tempe-rature (° C)	Snow-density (kg/m³)	Permeability (10^−10^ m^2^)	COV penetration resistance (N)	SD penetration resistance (N)	Snow tempe-rature (° C)	Snow-density (kg/m³)	Permeability (10^−10^ m^2^)	COV penetration resistance (N)	SD penetration resistance (N)	Snow tempe-rature (°C)
1	291	51.64	0.46	1.22	0.0	434	1.57	1.10	6.65	−1.1	434	5.51	0.24	2.75	−3.9
2	324	8.93	0.51	1.12	−1.5	402	3.83	0.75	6.18	−2.5	443	4.21	0.55	5.01	−4.2
3	354	11.00	0.67	1.09	−0.1	365	6.05	0.32	0.33	−0.8	439	6.38	0.48	3.95	−3.8
4	359	7.00	0.73	1.50	−0.1	395	3.01	0.47	2.96	−1.1	458	4.52	0.68	4.02	−3.2
5	363	19.11	0.58	1.99	−0.1	395	8.02	0.22	1.47	na	428	18.33	0.34	3.40	−4.9
6	381	5.10	0.84	1.15	−0.1	155	142.53	0.42	0.41	−1.3	425	6.12	0.36	2.40	−2.7
7	332	10.44	0.50	0.72	0.0	160	na	0.78	0.76	−0.7	443	9.53	0.42	3.77	−3.9
8	219	54.34	0.64	0.97	0.0	172	100.19	0.50	0.40	na	475	5.76	0.45	3.22	−4.2
9	321	34.59	0.60	1.37	0.0	204	45.01	0.46	0.53	na	452	5.07	0.47	4.25	−5.0
10	300	13.30	0.59	0.83	0.0	209	68.36	0.59	0.44	na	467	7.35	0.45	4.86	−2.4
11	286	31.43	1.04	1.49	0.0	159	35.18	0.49	0.38	na	481	4.58	1.05	11.33	−2.3
12	268	23.70	0.82	1.66	0.0	246	61.70	1.15	2.55	na	443	4.70	0.35	3.31	−2.8

na, value not available due to technical failure.

In the air pocket, O_2_ pocket decreased and CO_2_ pocket increased during each test and the linear relationship between them is shown in Fig. 3. Greater increases in CO_2_ pocket were observed along with marked decreases in O_2_ pocket at higher snow densities (upper left, Fig. 3). Lesser changes in gas composition witin the air pocket were mainly observed at intermediate/low snow densities (lower right, Fig. 3). Unexpectedly, substantial gas changes (mainly CO_2_ pocket) were also observed for low snow densities. Overall, SD of the penetration resistance was lower, when the snow density was low (i.e., a less permeable avalanche snow).

**Figure 2:**
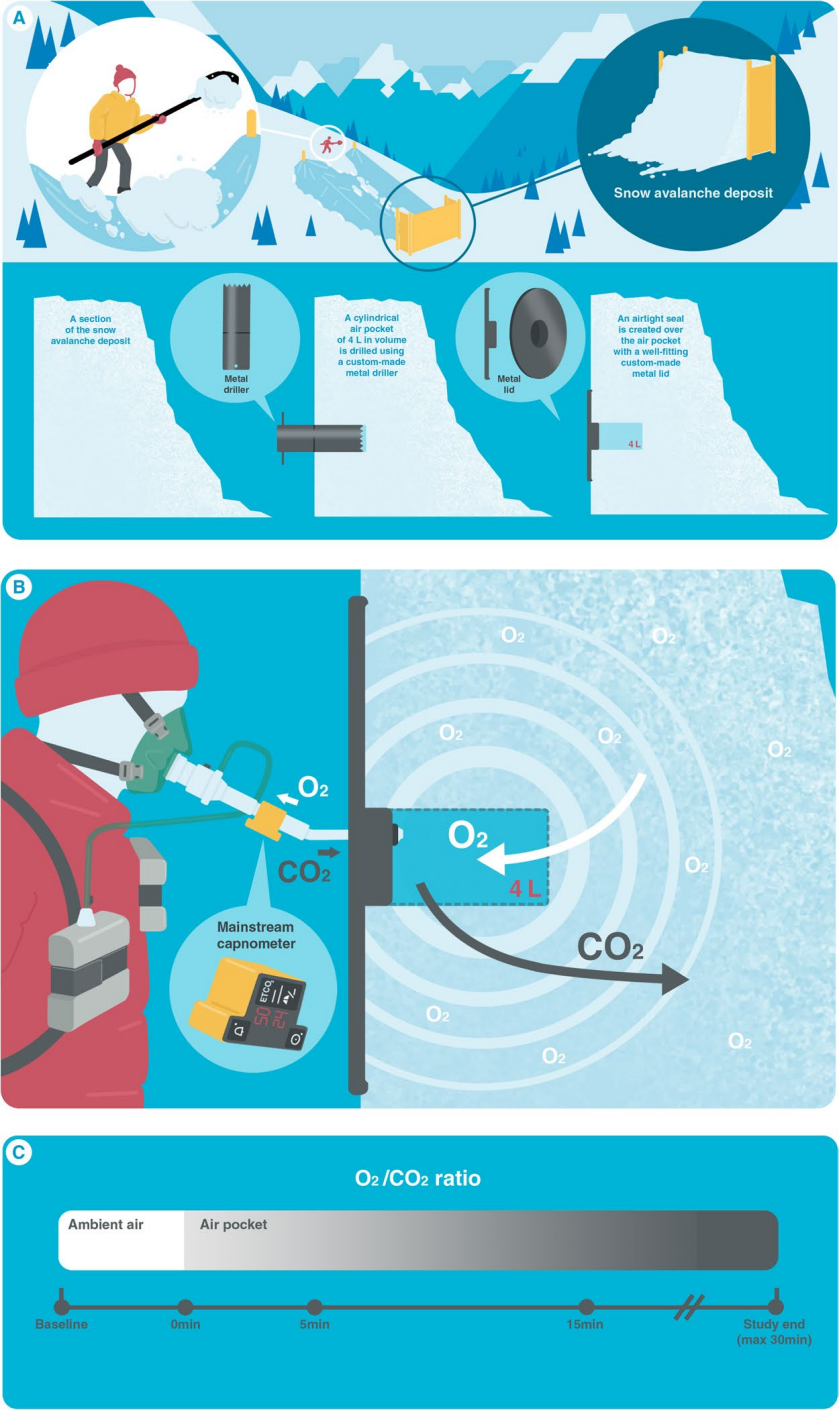
Study setting and data collection. (**A**) The snow was shoveled and transferred via a purpose built slide to create a snow deposit akin to that found in an avalanche scenario. A cylindrical air pocket of 4 L was drilled into the snow using a custom-made metal hole saw drill cutter. It had the form of a hollow tube with dentated open edge (Ø 15 cm). An air-tight seal was created over the air pocket with a well fitting custom-made metal lid. (**B**) The instrumented subject sat in front of the artificial avalanche. The subjects breathed into a closed system created by a face mask, the 20 cm long flexible plastic tube (Ø ~ 20 mm), the cylindrical air pocket and the surrounding snow. The porosity of the snow favored O_2_-diffusion from snow into the air pocket, whereas exhaled CO_2_ penetrated into the surrounding snow pack. (**C**) Data collection (for 36 study tests +6 contol tests): SpO_2_, etCO_2_, cardiac and air pocket monitoring together with spirometry were performed continuously (solid line) until interruption or end of experiment; capillary blood samples were collected at predetermined time-points (filled circles) and at interruption if test was suspended. Snow physical properties were collected during the baseline and after the study end of each test (vertical lines). (Illustration by Dalila Rovazzani. This figure is not covered by the CC BY licence. [Credits to copyright-holder by panel as appropriate]. All rights reserved, used with permission).

**Figure 3:**
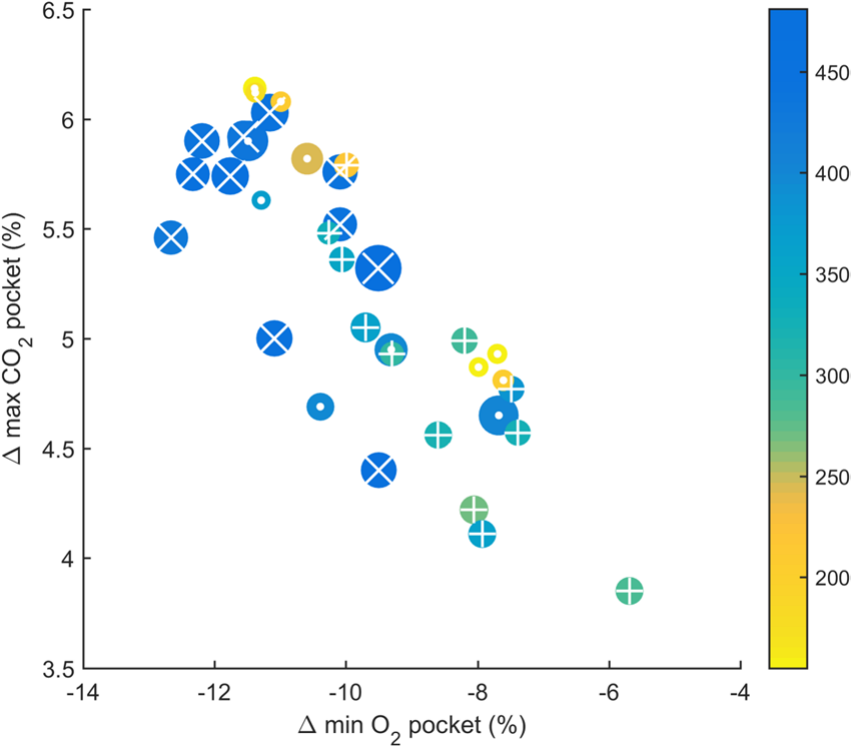
Changes of fractional concentration (%) of oxygen (O_2_) and carbon dioxide (CO_2_) during breathing experiments into the air pockets surrounded by snow with different properties (n = 35). Δ max indicates difference from baseline to maximum value and Δ min difference to minimum value. The colour bar indicates density (kg/m^3^) of the snow adjacent to the cavity. The area of the circles denotes the standard deviation (SD) of the penetration resistance 20 cm above and below the cavity. The markers in the circles indicate the measurement series: + (test series 1); ● (test series 2), and × (test series 3).

### Snow density clusters

Based on changes in O_2_ pocket and CO_2_ pocket, two clusters became clearly evident with respect to snow density. One cluster was formed by subjects breathing into an air pocket with low density and one for those with a higher density, with a possible linear correlation between snow density and differences in gas concentration (Supplementary Fig. 1). The existence of these two clusters was confirmed by a two-step cluster analysis, which identified the cases with a density ≤ 250 kg/m³ to be different from all other cases. It was therefore necessary to model snow density using two factors, one with the snow density itself and the other using an indicator having the value of one for cases with a density ≤ 250 kg/m³ and zero otherwise. Furthermore, the fixed scale subgrouping ( ≤ 250, 251–350, and > 350 kg/m³) was used for snow density where a subgroup analysis was needed. The steps to identify the snow density clusters are shown in the Supplementary materials, methods and results and Supplementary Fig. 1.

### Correlation of physical snow properties with changes in O_2_ and CO_2_ concentration in the air pocket

The wide range of changes in O_2_ pocket and CO_2_ pocket may be either due to individual human factors or due to the snow properties surrounding each cavity. In order to detect which snow physical properties were correlated with the O_2_-pocket decrease and the CO_2_-pocket increase, a general linear model with subject (volunteer) as a random factor was used. Only snow density (*p* < 0.001) and the indicator variable of a snow density ≤ 250 kg/m³ (*p* < 0.001) were correlated with O_2_-pocket values. There was no correlation of O_2_-pocket values with permeability (*p* = 0.725), snow temperature (*p* = 0.538) or COV (*p* = 0.650) and SD of penetration resistance (*p* = 0.674). Similarly, CO_2_-pocket values correlated with snow density (*p* < 0.001) and the indicator ≤ 250 kg/m³ (*p* < 0.001), but not with permeability (*p* = 0.777), snow temperature (*p* = 0.296), or COV (*p* = 0.177) and SD of penetration resistance (*p* = 0.613).

### Individual breathing behavior

**Figure 4:**
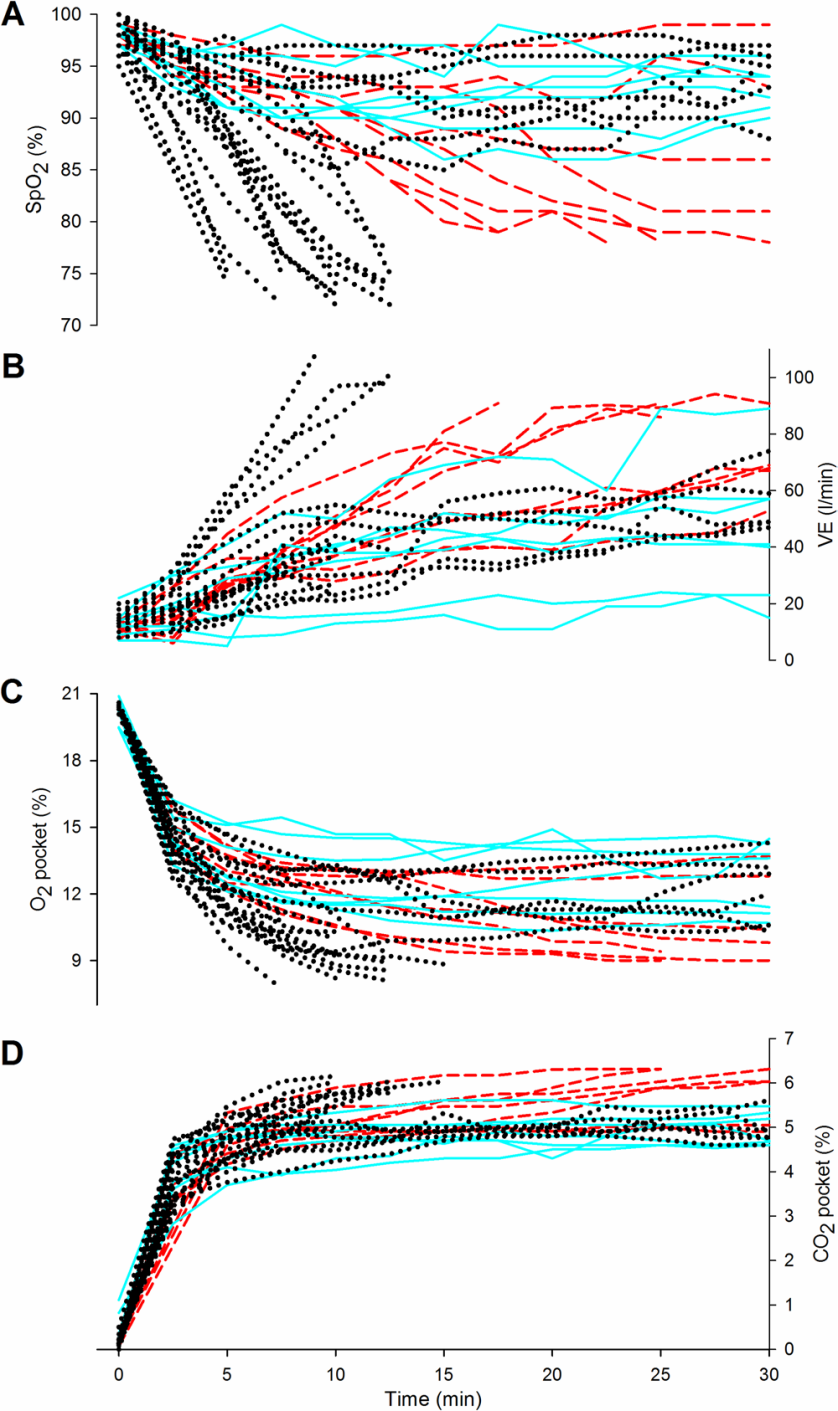
Curves of individual respiratory-gas and ventilatory parameters during the tests. (**A**) Peripheral oxygen saturation (SpO_2_). (**B**) Minute respiratory volume (VE). (**C**) Oxygen concentration in the air pocket (O_2_ pocket). (**D**) Carbon dioxide concentration in the air pocket (CO_2_ pocket). Dashed red line represents tests done breathing into an air pocket surrounded by snow density ≤ 250 kg/m^3^, solid blue line by snow density between 251–350 kg/m^3^ and dotted black line by snow density > 350 kg/m^3^ (n = 36 for **A**; n = 35 for **B,C** & **D**).

The measurement of individual ventilatory parameters (SpO_2_ and minute respiratory volume (VE)) and O_2_- and CO_2_-pocket concentration are shown, both for each test and snow density group in Fig. 4. Overall there was a progressive decrease in SpO_2_ and O_2_ pocket, with a parallel increase in VE and CO_2_ pocket. However, the individual ventilatory behavior between the three snow density groups was different and reportedly nonlinear in accordance to snow density, also within the same subject. Specifically, within the first 5 min (with data for all tests), the increase in VE differed (*p* = 0.017) between the three snow density groups. The increase in VE in the low and the high snow density groups were not different (*p* = 0.340), but there was a difference between the low and the intermediate snow density group (*p* = 0.047), plus a marginal but not significant difference between the intermediate and the high snow density group (*p* = 0.083). Within the first 5 min there was a marginal but not statistically significant difference between the snow density groups regarding the decrease in O_2_ pocket (*p* = 0.070), and no difference with regard to the increase in CO_2_ pocket (*p* = 0.145) and SpO_2_ (*p* = 0.491). Breathing, gas and circulation changes are given in the Supplementary results and Supplementary Fig. 2.

### Breath, gas and blood pressure factors

#### Principal Component Analysis (PCA)

Three factors grouping all parameter differences from baseline to minimum/maximum were identified (Table 3), which explain 75% of variance. The first factor may be called breath factor as it was positively correlated with breathing rate (BR), etCO_2_, heart rate (HR), tidal volume (VT) and VE. The second is the gas factor as it is positively correlated with O_2_ pocket, partial pressure of oxygen (pO_2_) and SpO_2_ and negatively with CO_2_ pocket, etCO_2_, HR and partial pressure of carbon dioxide (pCO_2_). The third factor is positively correlated with DBP and SBP and was therefore called blood pressure (BP) factor.

**Table 3:** Rotated component matrix and weight factors of principal component analysis (PCA). Δ max indicates difference from baseline to maximum value and Δ min difference to minimum value. Only weight factors greater than 0.4 or smaller than −0.4 are shown.

Variable	Breath factor	Gas factor	Blood pressure factor
Δ max VE	0.928		
Δ max VT	0.740		
Δ max BR	0.735		
Δ max HR	0.577	−0.496	
Δ min SpO_2_		0.850	
Δ min O_2_ pocket		0.905	
Δ min pO_2_		0.794	
Δ max pCO_2_		−0.796	
Δ max CO_2_ pocket		−0.707	
Δ max SBP			0.923
Δ max DBP			0.945
Δ max etCO_2_	0.612	−0.443	

BR, breathing rate; DBP, diastolic blood pressure; etCO_2_, end tidal CO_2_; HR, heart rate; pCO_2_, partial pressure of CO_2_; pO_2_, partial pressure of O_2_; SpO_2_, peripheral oxygen saturation; SBP, systolic blood pressure; VE, minute respiratory volume; VT, tidal volume.

#### Subgroups analysis

**Figure 5:**
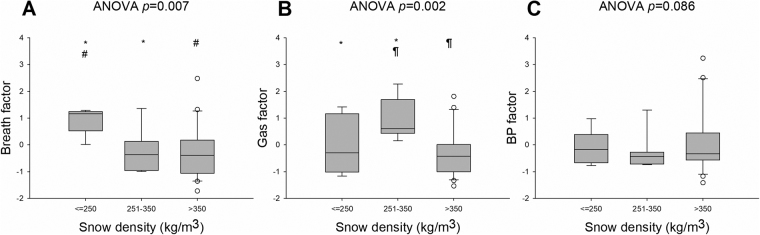
Boxplots of breath (**A**), gas (**B**) and blood pressure (**C**) factors per snow density group. *Indicates a significant difference between snow density groups ≤250 and 251–350 kg/m^3^, ^#^indicates a significant difference between snow density groups ≤250 and > 350 kg/m^3^ and ^¶^indicates a significant difference between snow density groups 251–350 and >350 kg/m^3^ (n = 36 for **A**,**B** & **C**). Blood pressure, BP.

Breath and gas factors showed overall a nonlinear relationship between the three different snow density groups (*p* = 0.007 and *p* = 0.002, respectively, Fig. 5). Specifically, for the breath factor the low snow density group demonstrated different mean values when compared to the intermediate and the high snow density group (*p* = 0.003 and *p* < 0.001, respectively). Conversely, for the gas factor the intermediate snow density group differed from the low and the high snow density group (*p* = 0.011 and *p* < 0.001, respectively). Thre was no significant difference for BP factor between the three different snow density groups (*p* = 0.086).

### Test duration

**Figure 6:**
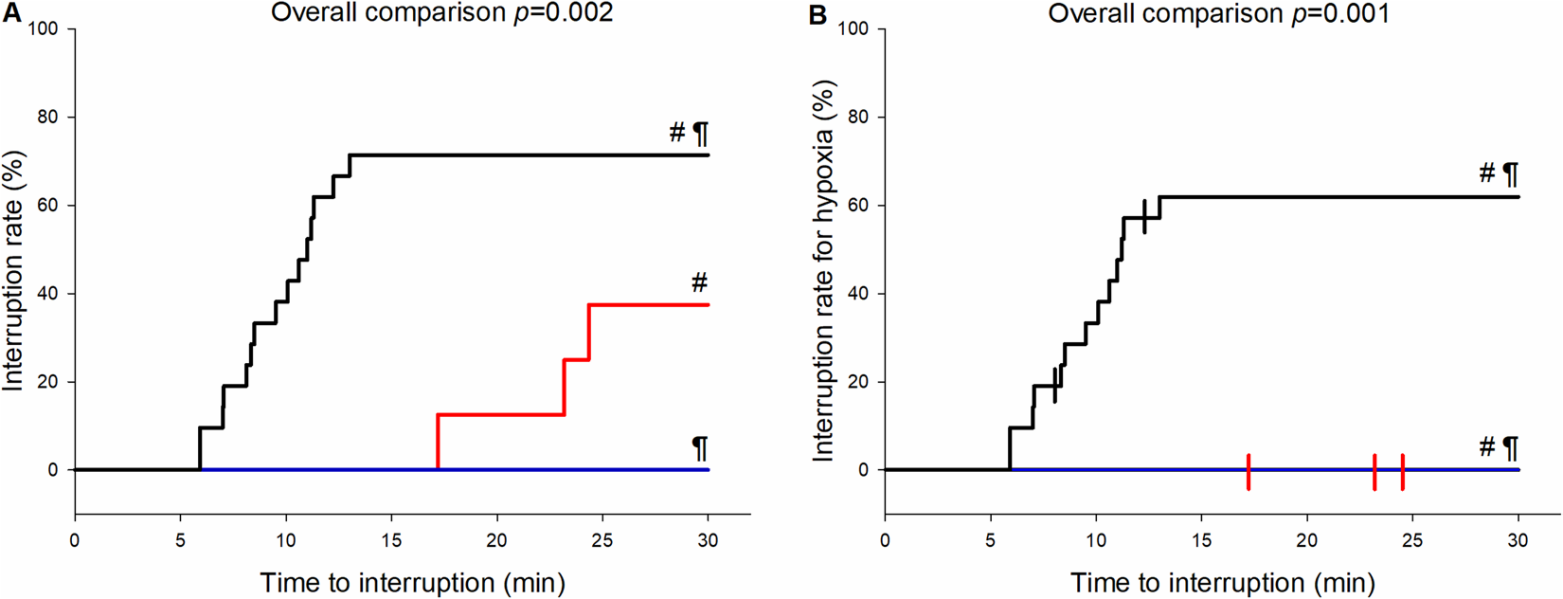
Test duration curves per snow density groups. (**A**) The curve considers all interruptions. (**B**) The curve shows only interruptions due to hypoxia (SpO_2_ < 75%). Red line represents snow density group ≤ 250 kg/m^3^, blue line snow density group between 251–350 kg/m^3^ and black line snow density group > 350 kg/m^3^; vertical markers in the right chart represent interruptions not due to hypoxia. ^#^Indicates a significant difference between snow density groups ≤ 250 and > 350 kg/m^3^ and ^¶^indicates a significant difference between snow density groups 251–350 and > 350 kg/m^3^ (n = 36 for **A** & **B**). Note that in the right chart blue and red line are overlapping.

Time to interruption differed between the three snow density groups (*p* = 0.002, Fig. 6) (study tests). Test interruption in the low snow density group was attributable only to clinical symptoms (dyspnea, headache, dizziness) and not due to hypoxia (SpO_2_ ≤ 75%). The two subjects who breathed into the air-tight 4 L control plastic bag (control tests) tolerated a median time of 196 s (range 170–228 s), and terminated the tests quicker than the subjects breathing into air pockets (*p* = 0.022 for each of the three test series) due to critical level of hypoxia (SpO_2_ ≤ 75%).

## Discussion

This study is the first to elucidate in detail the effects of snow properties on ventilation, oxygenation and exhaled CO_2_ in subjects breathing into an artificial air pocket. The properties of the snow surrounding the air pocket allow gas diffusion from the snow into the air pocket and vice versa. We found that a rapid decline in O_2_ and increase in CO_2_ were mainly associated with higher snow densities, whereas at lower densities (≤250 kg/m³), measured changes in gas concentrations were not as characteristic, indicating that the flow of CO_2_ out of the air pocket is not entirely reflected by snow density. Hence, snow density cannot serve as sole proxy for gas diffusion, but snow diffusivity and porosity need to be considered as well.

A clinical study design with integrated data-collection from human monitoring and snow-property analysis was developed. Specifically, we investigated our hypothesis in a comparable experimental model to that described previously by our group^7,8^ and others^6,9,12^; the study setting allowing safe and standardised conditions. During each experiment, subjects breathed into an air pocket of 4 L cut into artificially cultivated avalanche snow. SpO_2_ and O_2_-pocket concentration decreased progressively, with a parallel increase in CO_2_-pocket concentration. The decrease in SpO_2_ was much less than that observed in subjects breathing into an air-tight flexible plastic bag of the same volume, indicating a potential gas exchange while breathing into snow. Snow contains a considerable amount of interstitial air and assuming that the air entrapped in the snow is accessible by the subjects breathing into an air pocket, a larger (though unknown) air volume from the surrounding snow would be available to enable prolonged breathing. The gas diffusivity is, in fact, a known property of snow that indicates the ease with which gases can move through the interstitial pore space by diffusive processes^13^. The porosity of the snow surrounding the air pocket could favor O_2_-diffusion from snow into the air pocket where O_2_ is gradually consumed by the subject, whereas exhaled CO_2_ could diffuse in the opposite direction (Fig. 2).

The study results confirm our hypothesis that the time to the onset of critical levels of hypoxia and hypercapnia is influenced by snow properties. Time to interruption differed, in fact, between the three snow density groups. The larger interstitial air volume fraction in low compared to high-density snow suggests, in fact, that breathing into low-density snow allows a larger oxygen support than breathing into snow of a high density. However, the Fick’s law of gas diffusion doesn’t seem to be sufficient to fully explain the observed findings^13^. Avalanche snow is not uniform but highly irregular in terms of composition and density. In contrast to snow that is being transformed homogeneously in place in relation to time and climate, avalanche snow consists of rounded granules composed of aggregates of snow and ice particles (size in the dry and wet avalanches between 65 and 162 mm)^14^. The extent of this irregularity has been measured in this study by the variation in penetration resistance. Our hypothesis that this inhomogeneity would provide additional oxygen support was not confirmed, however. Although the variation in penetration resistance was low in the low-density group, we observed a progressive increase in CO_2_ at low snow density which seems to be independent from the low macro-permeability of the artificial avalanche deposit. This finding was supported by the lack of association between permeability (derived from micro-computed tomography (µCT))^15^ and the difference in O_2_- and CO_2_-pocket concentrations within a general linear model. Thus, the snow density (calculated as the mean of three density measurement methods, including µCT analysis)^16^, remains the only physical snow property associated with the pertinent observed gas changes in our experimental model. However, it remains unclear whether the increase in CO_2_ at low density is caused by a loss of CO_2_ absorption or by a diffusion barrier, attributable to the snow microstructure. Snow, in fact, forms a complex multiphase media inclusive of interstitial air, aerosol particles, an ice-air interface, and ice crystals^13^; a media highly vulnerable to metamorphism. Despite that there was no association in the present study with basal snow temperature, different gas diffusivity could be due to possible variation in adsorption and desorption processes, plus physical and chemical differences beween different molecules (O_2_ vs. CO_2_).

The concentration of the respiratory gas in the air pocket, plus the consequential impact on ventilatory control was different in accordance to the snow density and also present per subject. In general, the high snow density group demonstrated a rapid decrease in O_2_ pocket, plus a concomitant rapid increase in CO_2_ pocket that led to a progressive hypoxic milieu and a strong compensative ventilatory response (as shown by rapid increases of BR, VT, VE and etCO_2_), with a trend to premature interruption due to critical hypoxia (SpO_2_ ≤ 75%). Unexpectedly, the low snow density group showed a similar progression but with different timing and cause of interruption. The subjects of the low snow density group interrupted the test more often than expected, in all cases not due to hypoxia, but to a greater than expected increase in CO_2_ and the associated clinical symptoms (dyspnea, headache, dizziness). Due to the high number of measured parameters and their correlation, a Principal Component Analysis (PCA) approach was used to summarise the different respiratory-gas concentrations and ventilatory control in relation to the three groups of snow density. Two factors, the gas factor and the breath factor, were highlighted in the overall explaination of variance (58%). Although PCA analysis confirmed a similar inverse relationship in respiratory-gas concentrations within the air pocket, in both the high and low snow density groups, the ventilatory response was different. This differential response was presumably rather due to the rise in ventilatory drive in response to hypercapnia, than to hypoxia in the low snow density group. Hypercapnia is known to contribute to the sensation of dyspnea, and this may explain acute symptoms like headache and dizziness that prompted subjects breathing in the low snow density group to interrupt the test before reaching a SpO_2_ ≤ 75%. Hypercapnia per se has proved capable of inducing an increased inspiratory effort sensation to that reported at the same ventilation level during isocapnic conditions^17^. Whereas, the role of ventilatory resistance seems to be, at least with the air pocket volume used in the present study, minor as previously described in studies where smaller volumes were used (ranging from 0.06 to 2 L)^6,8^.

Previous studies have suggested that geographical and climatic factors may partially explain specific differences in survival in avalanche conditions. For example, in Western Canada, survival in maritime snow climates (i.e. high snow density due to mild temperatures) was characterised by a considerably earlier drop in survival compared to the curve for the continental snow climate (i.e. low snow density due to cold temperatures)^4^. This suggests an influence of snow density on survival. The current experimental results support this hypothesis and could have a direct impact on the refinement of international guidelines on both the management of avalanche patients^18^ and resuscitation guidelines, stating that after 60 min a patent airway is needed for long-term survival^19^. Subjects in the high snow density group could have a higher risk of normothermic cardiac arrest due to asphyxia (both due to a low concentration of O_2_ in the air pocket and hypercapnia-induced hypoxia). Conversely, it could be assumed that subjects breathing in an air pocket in low-density snow could develop a triple H syndrome (triad of hypoxia, hypercapnia and hypothermia)^7,8^. The prolonged time to the onset of a critical level of hypoxia, that may be preceded by early hypercapnia, could speed up cooling rate and favour the development of hypothermia (due to hypercapnia-induced unconsciousness, vasodilation and inhibition of shivering)^12^; plus a concomitant decrease in oxygen consumption of ~6% for every 1 °C reduction in core temperature^20,21^.

This study has some limitations. Firstly, the effect of cooling during snow burial on ventilation and gas exchange could not be investigated due to ethical reasons. However previously, in a similar study design where a porcine model was employed, the animals were rapidly cooled down to a mild-moderate level of hypothermia with a similar course of hypoxia and hypercapnia as demonstrated in this study; finally deteriorating further into asystole within 38 min. Each pig was positioned so that they breathed directly in an air pocket (1 or 2 L volume) of a comparable snow density to that of the high snow density group (i.e. > 400 kg/m³)^7^. Secondly, the study design aimed at maintaining a closed breathing-system to investigate the effect of different snow properties on an air pocket of the same gas volume. Validity and realiability of this model is supported by control tests and the lack of abnormal time series for single parameters (Fig. 4). However, the study design did not allow an extensive use of ergospirometric gas measurements. EtCO_2_ could be investigated only with a mainstream capnograph to assess the relationship between capillary and air pocket gas concentrations and SpO_2_. Thirdly, this study did not investigate the effect of breathing resistance. However, it can be assumed that flow resistance was limited, as shown in a study using a breathing system with a similar dead space and a smaller air pocket volume (0.06–1 vs. 4 L)^6^. Finally, despite the intention that this study was tailored to investigate the effect of snow density and other snow properties, the reason for the unexpected air-pocket gas concentrations in the low-density group cannot be fully elucidated as yet. We could speculate that potential snow metamorphism occurring during the tests may have influenced the results. The warm (37 °C) air expired into the air pocket may have changed the snow structure of the surface layer of the air pocket, affecting the air diffusion into the surrounding snow.

## Conclusions

Avalanche snow properties influenced ventilation and respiratory gases of subjects breathing into an artificial air pocket – suggesting that snow properties had a direct influence on ventilation, oxygenation and exhaled CO_2_. We found that a rapid decline in O_2_ and increase in CO_2_ were mainly associated with higher snow densities and led to premature interruptions due to critical hypoxia (SpO_2_ ≤ 75%). However, subjects in the low snow density group demonstrated a higher frequency of test interruptions than expected, due to clinical symptoms related to a rapid CO_2_ accumulation in the air pocket. These findings indicate that further investigation is necessary, as unknown factors related to physical snow properties, both inside and surrounding the air pocket, are responsible for the observed behavior in the low snow density group. Based on these results and in an attempt to better understand human breathing in air pockets with different snow densities, we suggest a laboratory study focusing on O_2_ and CO_2_ diffusion and adsorption/desorption in snow under controlled, artificial conditions analogous with avalanche snow burial of humans.

## Material and Methods

This randomized clinical trial was approved by the Institutional Review Board of the General Hospital of Bolzano (No. 0147248) and written informed consent was obtained from the subjects before enrollment in the study. All methods were performed in accordance with the relevant guidelines and regulations. The study registration number in the ClinicalTrials.gov register is NCT03082105 (last updated: March 10, 2017). The study was designed as a prospective and controlled randomized trial, using an age-matched group as control. All tests were performed in Prags, Dolomites, Italy (elevation 1499 m) between January and March 2014.

### Subjects

The sample included healthy Caucasian male volunteers (n = 14), 12 were enrolled into the snow air pocket group (study subjects) and two remained control subjects only. Prior to enrolment, all subjects were medically screened, including spirometry assessment, and classified according to the American Society of Anesthesiologists as ASA class I. Exclusion criteria were age < 18 years and any acute or chronic cardiovascular or respiratory disease.

In accordance with a randomization list, all subjects performed the test three times, in three test series, each separated by one month, i.e. January (test series 1), February (test series 2) and March (test series 3) 2014 (Fig. 1). The 12 subjects of the snow air pocket group were blinded with respect to the snow density.

### Experimental Setting

The experimental setting consisted of a snow pile with a vertical wall mimicking an artificial avalanche deposit (Fig. 2)^8^. The snow was collected on a slope above the study site, then shoveled onto a plastic slide and moved down the slope by approximately 25 m where it was piled against a wooden wall (about 3 m wide, 2 m high). After a short sintering time (about 30 min), the wooden panels were successively removed and for each subject a new cylindrical air pocket of 4 L in volume was cut out of the snow wall using a large, custom-made metal hole saw drill cutter in the form of a hollow tube with dentated open edge (Ø 15 cm). The resulting air pockets were sealed air-tightly with a well fitting custom-made metal lid with a highly compressed ring of moist snow on the external border. The procedure of creating an artificial avalanche deposit was repeated for each day of the study period. After instrumentation, the test subjects sat down to rest for approximately 30 min in front of the air pocket (outside of the snow, and protected from the cold with blankets, warm clothes and gloves) (Fig. 2). Thereafter the study period started. After fitting a Hans Rudolph oro-nasal face mask in an air-tight manner, subjects were breathing ambient air for 5 min (i.e. baseline) before the mask was connected to the artificial air pocket by a 20 cm long flexible plastic tube (Ø ~20 mm). The total additional dead space was 160 ml (99 ml mask + 60 ml tube). Test duration was scheduled for 30 min, but controlled with specific interruption criteria as follow: SpO_2_ ≤ 75%; hypercapnia (i.e. fractional inspired CO_2_ > 8%); at the subject’s request (e.g. due to subjective symptoms like dyspnea, dizziness, and headache), or any other worrying sign of cardiopulmonary or neurologic instability.

Prior studies with subjects breathing into artificial air pockets of only a 1 and 2 L volume resulted in a number of early interruptions^7,8^. Rapid development of hypoxia and a short test duration would have precluded us from investigating the influence of the snow density. As the real size of air pockets, that could surround the body surface (i.e. up to 1.81 m^2^ for a men with a height of 170 cm and a weight of 70 kg), is unknown so far, the 4 L air pocket volume was selected for this study. As control tests, in each one of the three test series two subjects breathed in an air-tight flexible plastic bag of a 4 L volume, simulating the ventilatory response of breathing into a sealed air pocket, where any interchange of gases from and to the surrounding snow is disabled.

### Measurements

#### Clinical parameters

The subjects were continuously observed and monitored by an emergency physician during the test period (Fig. 2). Non-invasive variables measured continuously included: blood pressure, 3-lead electrocardiogram, heart rate (HR) and SpO_2_ (Monitor HeartStart MRx^TM^, Philips Medical Systems, Andover, MA), breathing rate (BR), minute respiratory volume (VE) and tidal volume (VT) (Oxycon^TM^ mobile device, CareFusion Germany 234 GmbH, Hoechberg, Germany), and main stream end-tidal carbon dioxide (etCO_2_) (EMMA^TM^ Mainstream Capnometer, Masimo, Milan, Italy). Blood gases were measured from mixed capillary blood (ABL90 Flex^TM^, Radiometer, Copenhagen, Denmark; temperature corrected to 37 °C) at the beginning of the test, at 5 and 15 min, and at completion or interruption of the test before the disconnection of the tube and the air pocket.

#### Air pocket parameters

The fractional O_2_ and CO_2_ concentration in the air pocket was recorded continuously (X-AM 7000, Dräger, Vienna, Austria).

#### Snow parameters

Snow temperatures within the artificial avalanche deposit were measured initially and subsequently after each experiment near the surface and both 5 and 10 cm from the artificial air pocket.

Snow density was measured at three different scales. First, snow density was determined by weighing the snow volume cut out to create the artificial air pocket (i.e., bulk density). Second, multiple snow samples were taken from the sidewall of the cavity after the breathing experiments with a 100 cm^3^ density cutter and weighed with an electronic scale to determine density^16^. Third, additional small samples were taken from the sidewall after the breathing experiments and transported on dry ice to the cold laboratory at the WSL Institute for Snow and Avalanche Research SLF, Davos, Switzerland. There, samples of about 3 cm^3^ were scanned by micro-computed tomography (µCT)^22^, plus the three dimensional microstructure was reconstructed to derive the ice volume fraction, yielding the snow density^16^. Values from the three density measurement methods (representing different scales) were correlated. Hence, for analysis, the bulk density, the mean cutter density and the mean µCT density were averaged. The µCT analysis allowed the derivation of additional snow microstructural properties, such as the permeability. The permeability of snow can be derived from the specific surface area of snow grains and snow density from µCT analysis^15^. To assess the variability of physical snow properties in the artificial avalanche deposit, snow micro-penetrometer (SMP) measurements were also completed with SnowMicroPen^23^. From these vertical SMP profiles, taken through the center of the artificial air pocket, the penetration resistance (including its variation around the cavity) was derived post each breathing experiment. The standard deviation (SD) and the coefficient of variation (COV) of the penetration resistance, both 20 cm above and below the cavity, was used in the subsequent data analysis.

### Statistical Analysis

The sample size estimation was calculated based on pO_2_ assuming an effect size (mean difference/common standard deviation) of 1, a power of 80% and an overall significance level of 5%, with an assumed dropout rate of 20%. It resulted to be n = 14 subjects (12 study subjects and two control subjects) per session. The subjects were randomised per test series blocked by session. Difference from baseline to maximum value of breathing rate (BR), CO_2_-pocket concentration, diastolic blood pressure (DBP), etCO_2_, pCO_2_, HR, systolic blood pressure (SBP), VE and VT, and difference from baseline to minimum value of pO_2_, SpO_2_ and O_2_-pocket concentration were considered as variables for analysis; they were visibly checked for normality by means of normal probability plot. A Principal Component Analysis (PCA) with Varimax rotation was performed in order to reduce the number of parameters and to check the reliability of the data; eigenvalues > 1 were used to identify the number of factors. A two-step cluster analysis was used to identify groups of snow density. This analysis included the factors snow density and the residuals of a linear regression of snow density and the concentration of O_2_- and CO_2_-pocket values, using only cases with densities > 347 kg/m³ (mean snow density) for estimating the linear model. A general linear model with subject as random factor was performed to investigate correlation of snow physical properties (i.e., density, permeability, snow temperature, coefficient of variation of penetration resistance and standard deviation of penetration resistance) with changes in O_2_- and CO_2_-pocket concentrations. Snow density was included, using the snow density itself, plus and indicator for cases with a density ≤ 250 kg/m³. General linear models, with subject as random factor, were also used to compare groups of snow density by means of ANOVA and *p*-values of pairwise comparisons were then corrected by means of Holm-Bonferroni. Test duration was analysed by means of the Kaplan-Meier estimator and log-rank tests for group comparisons. Additionally, a Mann-Whitney *U* test was performed to compare test duration between the intervention and control group. SPSS version 23.0 statistical software (IBM Corp., Armonk, NY) was used. Tests were two-sided and *p* < 0.05 was considered statistically significant. Values are reported as mean ± standard deviation unless stated otherwise.

### Data availability

The data that support the findings of this study are available from the corresponding author on reasonable request.

## Electronic supplementary material

Supplementary material
