# Supplementary material for: Effects of snow properties on humans breathing into an artificial air pocket – an experimental field study
Source: Sci Rep. 2017 Dec 15;7:17675. doi: 10.1038/s41598-017-17960-4 (PMC5732296; doi:10.1038/s41598-017-17960-4)

**Effects of snow properties on humans breathing into an artificial air pocket – an experimental field study**

Giacomo Strapazzon^1*^, Peter Paal^2,3^, Jürg Schweizer^4^, Markus Falk^1^, Benjamin Reuter^4^, Kai Schenk^1,5^, Hannes Gatterer^5^, Katharina Grasegger^1^, Tomas Dal Cappello^1^, Sandro Malacrida^1^, Lukas Riess^6^, Hermann Brugger^1,6^

^1^ Institute of Mountain Emergency Medicine, Eurac Research, Bolzano, Italy

^2^ Department of Anaesthesiology and Intensive Care Medicine, Hospitallers Brothers Hospital, Teaching Hospital of the Paracelsus Medical University Salzburg, Salzburg, Austria

^3^ Department of Perioperative Medicine, Barts Heart Centre, William Harvey Research Institute, Barts & The London School of Medicine&Dentistry, Queen Mary University of London, London, UK

^4^ WSL Institute for Snow and Avalanche Research SLF, Davos, Switzerland

^5^ Department of Sport Science, Medical Section, University of Innsbruck, Innsbruck, Austria

^6^ Department of Anaesthesiology and Intensive Care Medicine, Medical University Innsbruck, Innsbruck, Austria

* Corresponding author: Giacomo Strapazzon, MD PhD. Institute of Mountain Emergency Medicine, Eurac Research, Viale Druso 1, 39100 Bolzano, Italy; Email [giacomo.strapazzon@eurac.edu](mailto:Giacomo.strapazzon@eurac.edu); Tel.: +39 0471 055543; Fax.: +39 0471 055549.

**Running head:** Snow properties and human breathing

**Conflict of interest:** No conflict of interest exists for any author

**Funding:** This study was supported by internal funding only

**Supplementary materials, methods and results**

*Cluster analysis*

The scatterplot of the difference from baseline to the maximum concentration of CO_2_ pocket, in relation to the difference from baseline to the minimum concentration of O_2_ pocket (with respective subgroups according to mean snow density) is shown in the left chart of Supplementary Fig. 1. Regression lines of given subgroups are different (p = 0.016, indicator of density above the mean of 347 kg/m³ in GLM). Assuming that the effect of snow density is linear and identical between subgroups, an estimated regression model for one subgroup should be valid for both subgroups. We have therefore performed a regression analysis on O_2_– and CO_2_–pocket concentration with resepect to snow density using only cases with a snow density above the mean of 347 kg/m³. For all cases the respectively estimated standardised residuals of O_2_– and CO_2_–pocket concentrations were supplied to a two-step cluster analysis together with snow density and two subgroups were identified by using an automatic cluster selection with Schwarz’s Bayesian Criterion. The two scatterplots (right charts of Supplementary Fig. 1) highlight these two clusters: one consisting of eight cases (snow density ≤250 kg/m³), demonstrating a larger increase in CO_2_–pocket concentration and respective decrease in O_2_, with respect to what would have been expected according to that snow density. As the influence of snow density in the second cluster with regard to gas concentration value is linear, we decided to use a fixed scale to subdivide this cluster into three groups. Thus forming four groups in total with respect to snow density: ≤250 kg/m³ (eight cases), 251-350 kg/m³ (seven cases), 351-450 kg/m³ (16 cases) and >450 kg/m³ (five cases). As a log-rank test showed a difference in test duration between these four groups (*p*=0.004), but not between the groups with a snow density of 351-450 kg/m³ and >450 kg/m³ (*p*=0.479), plus in the latter group there were only five cases, the two groups were combined into a single group. Hence, three groups of snow density were considered for analysis: ≤250 kg/m³ (eight cases), 251-350 kg/m³ (seven cases), and >350 kg/m³ (21 cases).

*Subgroup analysis of single parameters of differing snow density groups*

Further details regarding the non-linear dependency of the investigated parameters with respect to snow density are depicted in Supplementary Fig. 2 and 3. The intermediate snow density group (251-350 kg/m³) demonstrated more favorable values in numerous parameters in comparison to the other two groups (low and high); particularly with respect to higher oxygenation (*p*=0.048 and *p*<0.001 in comparison with low and high snow density groups for difference from baseline to minimum SpO_2_, respectively; *p*=0.040 and *p*=0.001 in comparison with low and high snow density groups for difference from baseline to minimum O_2_ pocket, respectively) and lower hypercapnia (*p*=0.003 in comparison with both low and high snow density groups for difference from baseline to maximum pCO_2_; *p*<0.001 and *p*=0. 001 in comparison with low and high snow density groups for CO_2_ pocket, respectively). Although the difference from baseline to maximum HR and SBP values showed a similar measurement pattern and were reportedly different between the three groups (*p*=0.030 and *p*=0.040, respectively), no significant pairwise comparison was detected. Conversely, differences from baseline to maximum VE values recorded in the low snow density group were different from the other two groups (*p*=0.010 for comparison with 251-350 kg/m³ snow density group and *p*=0.008 for comparison with >350 kg/m³ snow density group), however there was no significant difference between the intermediate and the high snow density group (*p*=0.413). The difference from baseline to maximum etCO_2_ values demonstrated only a difference between the low and the intermediate snow density groups (*p*=0.044). There was no difference between snow density groups in regard to difference from baseline to minimum of pO_2_ (*p*=0.219), nor difference from baseline to maximum of BR (*p*=0.423), DBP (*p*=0.074) and VT (*p*=0.054).

**Supplementary figures**

**Supplementary Figure 1.** On the left (**A**), a scatterplot of difference from baseline to maximum (Δ max) CO_2_–pocket concentration, in relation to difference from baseline to minimum (Δ min) O_2_–pocket concentration with regression line. Green dots represents snow density values under the mean of 347 kg/m^3^, whereas blue dots represents those values above the mean; the regression line for all cases is in black. On the right (**B** & **C**), scatterplots depicting the difference from baseline to maximum CO_2_ pocket, plus the difference from baseline to minimum O_2_ pocket, in relation to snow density with regression lines. Grey represents snow density ≤250 kg/m^3^ and black represents values >250 kg/m^3^ (n=35 for A, B & C).

**
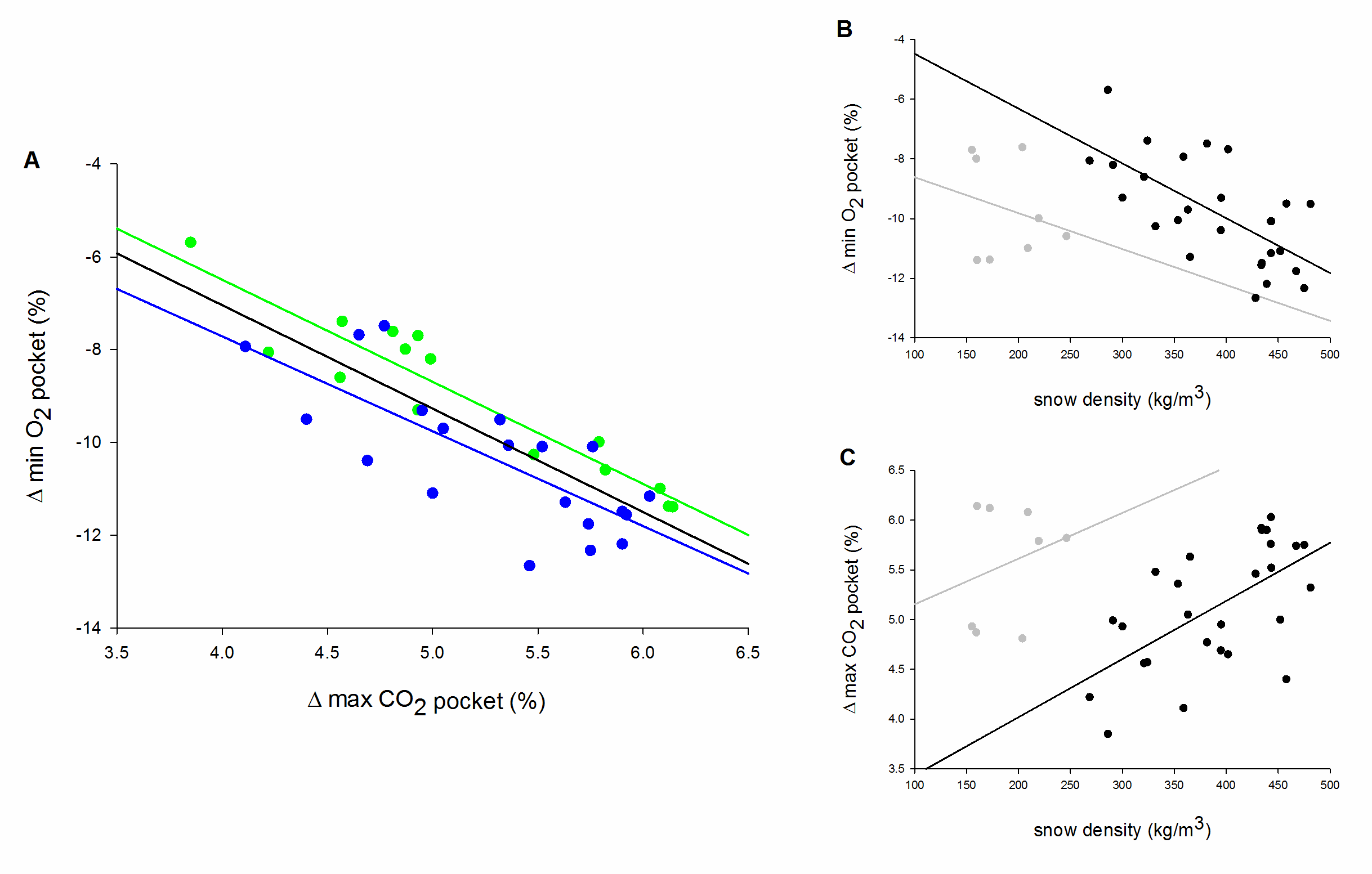
**

**Supplementary Figure 2.** Boxplots of the difference from baseline to maximum value (Δ max) for VE, BR, VT, etCO_2_ and HR, plus for difference from baseline to minimum value (Δ min) SpO_2_ per snow density group (n=36 for A, C & F; n=35 for B & D; n=34 for E). * indicates a significant difference between ≤250 and 251-350 kg/m^3^, # indicates a significant difference between ≤250 and >350 kg/m^3^ and ¶ indicates a significant difference between 251-350 and >350 kg/m^3^. BR, breathing rate; etCO_2_, end tidal CO_2_; HR, heart rate; SpO_2_, peripheral oxygen saturation; VE, minute respiratory volume; VT, tidal volume.

**
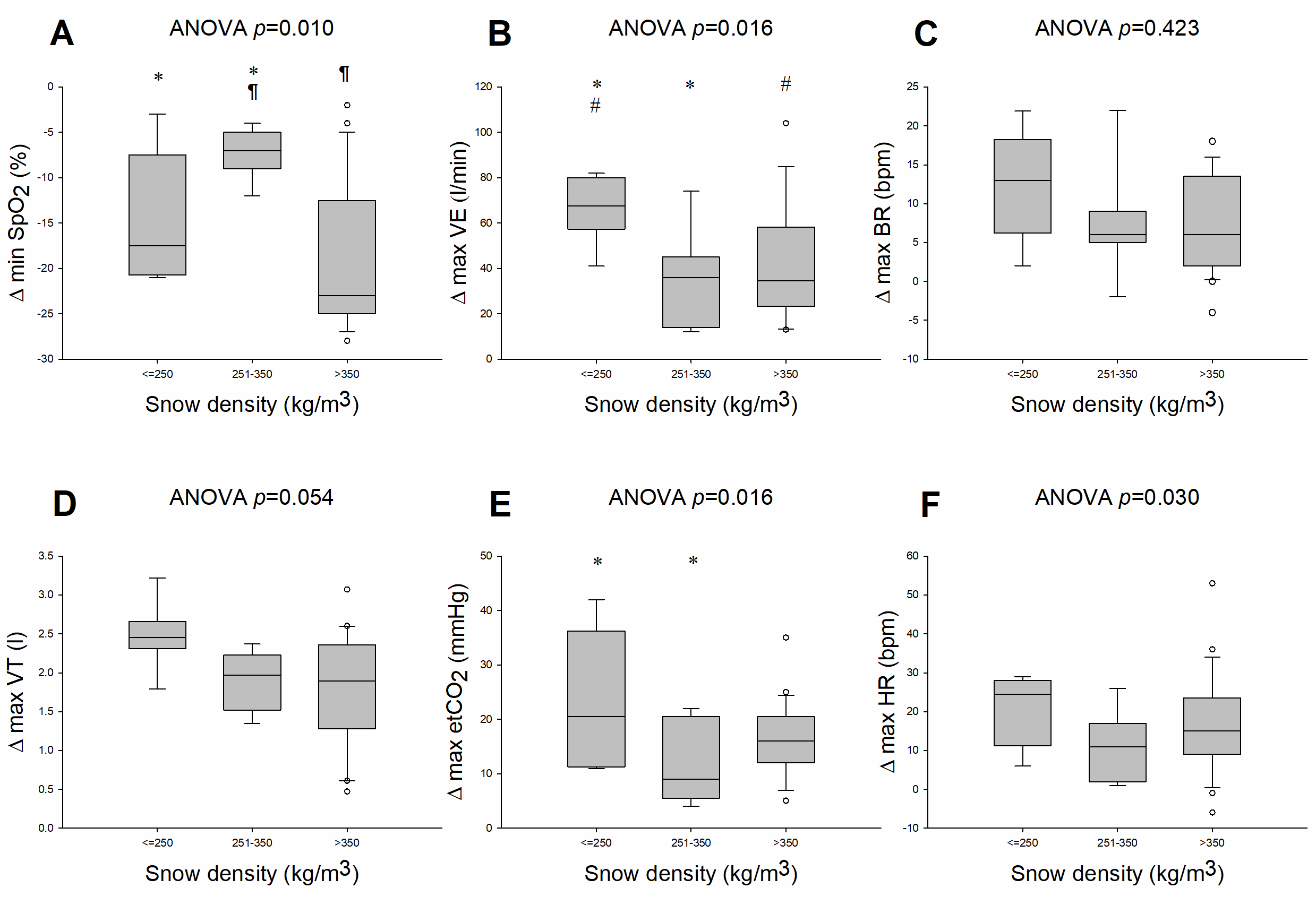
**

**Supplementary Figure 3.** Boxplots of the difference from baseline to maximum value (Δ max) CO_2_ pocket, pCO_2_, SBP and DBP, plus the difference from baseline to minimum value (Δ min) of O_2_ pocket and pO_2_ per snow density group (n=35 for A, B & C; n=36 for D, E & F). * indicates a significant difference between ≤250 and 251-350 kg/m^3^, # indicates a significant difference between ≤250 and >350 kg/m^3^ and ¶ indicates a significant difference between 251-350 and >350 kg/m^3^. DBP, diastolic blood pressure; pCO_2_, partial pressure of CO_2_; pO_2_, partial pressure of O_2_; SBP, systolic blood pressure.


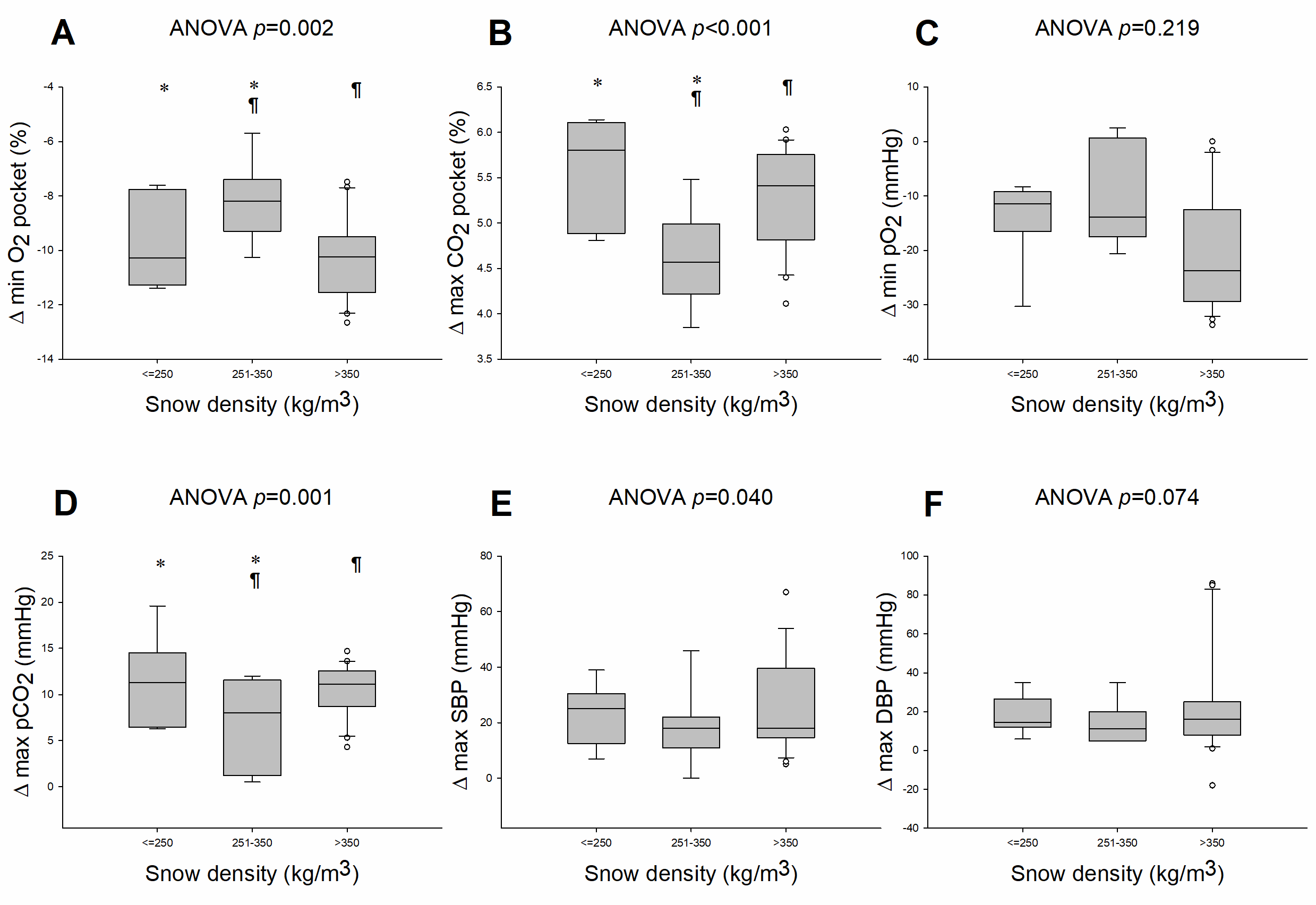

Supplement: Supplementary file 1 — Supplementary material [file 41598_2017_17960_MOESM1_ESM.doc]
